# Voluntarily stopping eating and drinking (VSED) to hasten death: may clinicians legally support patients to VSED?

**DOI:** 10.1186/s12916-017-0951-0

**Published:** 2017-10-20

**Authors:** Thaddeus Mason Pope

**Affiliations:** Mitchell Hamline School of Law, 875 Summit Avenue, Saint Paul, Minnesota 55105 USA

**Keywords:** Voluntarily stopping eating and drinking, Medical aid in dying, Assisted suicide, Suicide, Law, End-of-life, Exit option

## Abstract

Jox and colleagues recently compared and contrasted two leading end-of-life exit options, namely voluntarily stopping eating and drinking (VSED) and medical aid in dying (MAID). The authors argue that policymakers and medical societies should consider VSED and MAID in a uniform and consistent manner given that clinician participation in both constitutes assisted suicide. This is a very controversial topic. Herein, it is questioned whether there really is disparate consideration of VSED and MAID and whether it is justified, bearing in mind that VSED is not assisted suicide.

Please see related article: http://bmcmedicine.biomedcentral.com/articles/10.1186/s12916-017-0950-1.

## Background

The article by Jox et al. [1] is a most welcome contribution to the literature on the ethical and legal justifiability of medically supervised end-of-life exit options. Medical aid in dying (MAID) has been the subject of extensive academic discussion, widespread legislative activity, and voluminous court litigation. In contrast, voluntarily stopping eating and drinking (VSED) has been the subject of very little academic attention and almost zero legislative or judicial activity [2]. Such silence and neglect are unfortunate given the uncertainty they create with regards to the ethical or legal status of VSED. However, considering that VSED is a potentially valuable exit option, providing clarity on these issues is paramount. Indeed, for many patients, VSED may be the only available exit option since MAID remains illegal in most jurisdictions and, even when it is authorized, it is usually limited to patients with capacity.

Jox et al. [1] do not defend the legitimacy of either VSED or MAID. Instead, they argue that clinicians and policymakers should consider VSED and MAID (whatever the ethical and legal status of those options) in a uniform and consistent manner, providing only two choices – to either allow or deny both. The authors defend their argument in two stages. First, they contend that law and medical practice often consider VSED and MAID differently, typically permitting VSED and prohibiting MAID. Second, they contend that this distinction is unwarranted because clinician participation in VSED constitutes assisted suicide as equally as it does in MAID.

Herein, I question both premises in support of this argument. First, it is unclear whether law and medical practice really do consider VSED and MAID differently. Second, even if such a distinction between VSED and MAID indeed occurs, this difference is justified since clinician participation in VSED does not constitute assisted suicide.

## Inconsistent consideration of VSED and MAID

Jox et al. [1] state that palliative care organizations “*are increasingly advocating*” and have a “*widely held position*” to “*approve and even promote*” VSED. Further, they write that “*most Western jurisdictions seem to permit medical support for VSED*”. For three reasons, I argue that the authors misstate, or at least overstate, the disparate consideration of VSED and MAID.

First, there is remarkably little support for VSED from professional medical associations. Admittedly, they do not formally oppose VSED in the way that MAID is usually opposed. However, the absence of opposition does not indicate support. Almost no medical association, either in the United States, Europe, or elsewhere, has formally supported or even taken a neutral position on VSED [3]. Indeed, Jox et al. [1] cited just two European medical associations, whereas the American Nurses Association just recently published a favorable position statement [4]. In short, medical societies are not ‘advocating’ VSED but are instead mostly just ignoring it.

Second, courts and legislatures have been as silent as medical associations, without the audible granting of permissions. Admittedly, there has never been a reported criminal prosecution or medical board discipline action related to VSED; yet, this does not equate to a permission. Thus, the legal status of VSED remains unclear, with negligible judicial precedent, legislative guidance, or regulatory direction [5, 6].

Third, it is difficult to demonstrate inconsistent handling of VSED and MAID given the constant flux in the management of MAID (especially in North America). Medical associations, legislatures, and courts have increasingly (and rapidly) recognized the legitimacy of MAID [7–9]. Consequently, even if Jox et al. [1] are correct about the (positive and growing) support for VSED, they have misconceived the lack of support for MAID. In short, it is difficult to compare the medical and legal consideration of VSED and MAID in a categorical and absolute fashion because each exit option is itself regarded in highly variable ways across jurisdictions, institutions, and professional societies.

Notwithstanding these three points, there is at least some disparate consideration between VSED and MAID. For example, seven U.S. jurisdictions explicitly and affirmatively authorize MAID [10]. Yet, none of these states expressly authorizes VSED. Similarly, several U.S. states explicitly and affirmatively prohibit MAID. Yet, none of these states expressly prohibits VSED. As Jox et al. [1] observe, “*a clear legal basis for medically supported VSED in statute or common law is often lacking*”. In short, VSED and MAID do not differ due to the acceptance of one and rejection of the other, but rather due to the clear legal status of MAID and the ambiguity in that of VSED.

## Law is defined by experience, not logic

In the second stage of their argument, Jox et al. [1] argue that law and medical practice should consider VSED and MAID equally given that VSED constitutes assisted suicide in all the relevant ways that clinician participation in MAID does. This argument is a useful prompt for reflection. Yet, it fails as a call for policy reform because it wrongly assumes that semantics is a sufficient basis for legal argument. As U.S. legal scholar and judge, Oliver Wendell Holmes, famously observed: “*The life of the law has not been logic; it has been experience. The law embodies the story of a nation’s development through many centuries, and it cannot be dealt with as if it contained only the axioms and corollaries of a book of mathematics*” [11].

Deducing policy conclusions from a purely semantic analysis produces absurd results. Every minute of every day, patients decide to stop dialysis, to cease mechanical ventilation, or to refuse artificial nutrition and hydration. Clinicians comply with these requests despite knowing that they will lead to patient death. Conceptually, withholding or withdrawing life-sustaining treatment is ‘assisted suicide’ , yet neither law nor ethics considers (or is prepared to consider) them so.

Furthermore, Jox et al. [1] seem to apply conceptual semantics to a legal or policy argument without considering many of the existing moral and cultural norms. The authors concede that VSED is not typical suicide because there is “*no invasive or aggressive act*”. Yet, they dismiss this as insignificant, ignoring the widely accepted distinction between active and passive means of hastening death [12]. Active methods like MAID, which entail the introduction of a lethal agent, are generally prohibited; however, passive means like VSED, which entail only the refusal of an intervention, are generally permitted. This active/passive distinction may not be philosophically sound, but it has been engrained in law and ethics for decades. Thus, in short, given their differing passive and active nature, there are ample and adequate grounds for the disparate consideration of VSED and MAID [13].

## Conclusion

Jox et al. [1] are right to call on policymakers and professional societies to promote a critical, evidence-based and transparent discussion of VSED. Moreover, their article may usefully prompt this much-needed deliberation. While it remains unclear whether we should harmonize laws and policies concerning VSED and MAID, it is imperative that we clarify the ethical and legal status of VSED.
